# Effect of Comprehensive Surgical Safety System on Patients’ Outcome: A Prospective Clinical Study

**DOI:** 10.7759/cureus.2601

**Published:** 2018-05-10

**Authors:** Nishkarsh Mehta, Anandhi Amaranathan, Loganathan Jayapal, Pankaj Kundra, Vishnu Prasad Nelamangala Ramakrishnaiah

**Affiliations:** 1 Surgery, Jawaharlal Institute of Postgraduate Medical Education and Research (JIPMER), Puducherry, IND; 2 Department of Anesthesiology, Jawaharlal Institute of Postgraduate Medical Education and Research (JIPMER), Puducherry, IND; 3 HPB Surgery, Jawaharlal Institute of Postgraduate Medical Education and Research (JIPMER), Puducherry, IND

**Keywords:** surgical patient safety system, post operative outcome

## Abstract

Background

Patient safety has become an integral part of hospital management to prevent catastrophic events which adversely affects the patients, care providers and the hospital. Surgical Checklists are an easy and simple way to prevent surgical errors and complications.

Objective

This prospective study is to evaluate the effect of SURPASS (Surgical Patient Safety System) checklist on the outcome of the patients who underwent surgery in our hospital.

Methods

All the patients who underwent surgery in the sixth unit of Department of Surgery from April 2014 to May 2015 were included in the study excluding those aged above 13 years and day care surgery cases. For the control group (initial six months) no checklist was implemented whereas for the study group (next six months) SURPASS checklist was implemented. Data collected on age, sex, diagnosis, surgical procedure, type of anaesthesia, number and type of postoperative complications, need of second surgery because of complications, length of hospital stay and outcome (discharge, disability or death). Mann–Whitney U test and Fisher’s exact test were used for analysis.

Results

Of the total 372 patients operated, 200 were before and 172 were after implementation of SURPASS checklist. Before implementation of the checklist, complications were noticed in 66.66% of elective and 77.23% of emergency cases. Whereas after implementation of checklist the complications in elective cases were found to be 51.09% (p-value = 0.008) and 67.50% (p-value = 0.024) in emergency cases.

Conclusion

Implementation of SURPASS checklist is effective in reducing the rate of postoperative complications in both elective and emergency surgeries.

## Introduction

Patient safety has become an integral part of hospital management to prevent catastrophic events which adversely affect the patients, care providers and the hospital as a whole. Though such events are known to occur in all the patients, it is estimated that almost two-thirds of such events are observed with surgical care [1].

Since the incidence is more in relation to the surgical patients, several measures have been proposed to increase the safety of surgical patients such as training the entire team involved in the patient management, imparting teamwork spirit in the operating room and the most important being the introduction of the surgical checklist. Surgical Checklists are an easy and simple way to prevent surgical errors and complications. After starting of checklist era, many checklist systems developed worldwide. Most surgical safety interventions and checklists have focused only in the operating room and not outside the operating room. But a significant number of surgical errors (30-70%) occur outside the operating room (pre-operative and post-operative) [2-4]. These results made the concept that improvement in patient safety can be made by taking care of full surgical pathway.

This leads way to the development of a multidisciplinary SURPASS (Surgical Patient Safety System) checklist which follows the surgical pathway from the admission of the patient to the hospital till they get discharged [2].

SURPASS checklist is the first valid checklist for the entire surgical pathway. It was developed and implemented by de Vries et al. in 2009 in the Netherlands. They developed, validated and evaluated a Surgical Patient Saftey System (SURPASS) checklist [2]. The list is multidisciplinary and is completed by different members of the team such as ward doctor, surgeon, anesthesiologist, operation room assistant, and nurse. This checklist covers entire surgical pathway of the patient from admission to discharge including operative room also. It is divided into different stages (preoperative ward, operating room, recovery or intensive care unit, postoperative ward) and focused on all movements of the patient (including admission and discharge).

A considerable amount of damage, both physical and financial, is likely to be prevented by using the SURPASS checklist [5]. We did this study to evaluate the effect of SURPASS checklist on patients outcome in our hospital.

## Materials and methods

This prospective clinical study was carried out in the Department of Surgery, in a tertiary care hospital, India from April 2014 to May 2015. Postgraduate research monitoring committee and Institute Human Ethics Committee (IEC) approval were obtained to conduct the study.

All patients operated (electively/emergency) in a single surgical unit of Department of Surgery from April 2014 to May 2015 were included in the study. Patients whose age was less than 13 years or underwent surgery under local anesthesia and those who got discharged within 24 hours of admission were excluded from the study. Patients were divided into two groups.

The first group (control group)

During the initial six-month period, after admission to the hospital, all basic and demographic data, diagnosis and surgical procedure underwent by the patient were noted. All patients were followed up after surgery till they got discharged from the hospital and during this time if the patient developed any complication it was noted.

The second group (study group)

**Figure 1:**
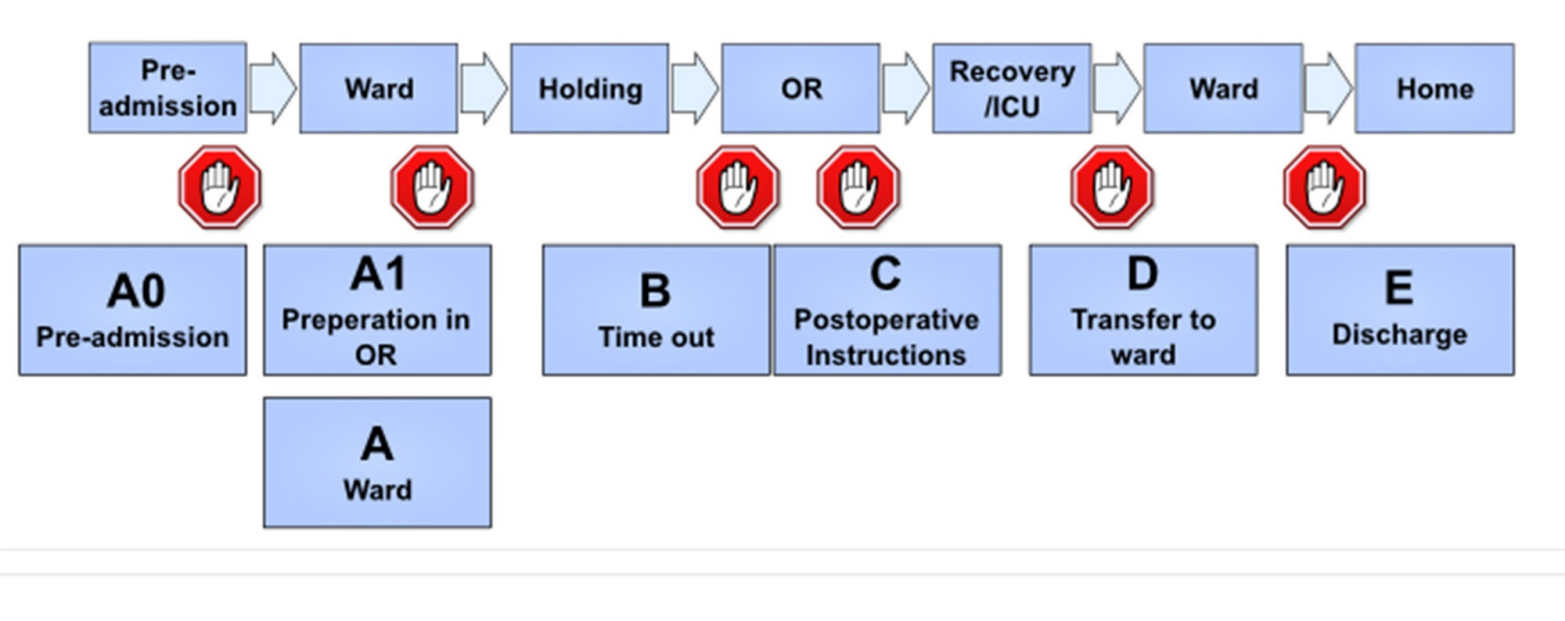
Surgical patient safety system.

During the next six months of the study, SURPASS checklist was implemented (Figure 1). During this period, patients were followed up from admission till discharge according to the checklist as (a) preoperative in the ward, before shifting to operation theatre, in the recovery room, after recovery room to the ward or intensive care unit (ICU), the postoperative period in the ward and discharge. All the items in the checklist were checked by the responsible person before proceeding to the next step and interventions and arrangements of things done accordingly. During postoperative course, complications related to the surgery were noted. During study level of compliance was more than 90%.

The outcome of the patients was noted in the form of discharge, permanent disability or death. After completion of the study, results of both the groups in the form of the numbers of postoperative complications and the outcome of the patients were compared and statistical analysis was done.

Data collection

Data were collected on age of the patients (in years), sex, system involved, surgical procedure, type of anaesthesia (general or regional anaesthesia), postoperative complications, number and type of complications, need of second surgery, length of hospital stay (in days), outcome – discharge, disability or death.

All postoperative complications were registered by treating team of doctors during the patient’s hospital stay. Complications that developed after discharge were not documented.

Statistical analysis

All recorded complications were classified according to system involved. Differences between patients undergoing surgery before and after implementation of the checklist were assessed with the use of the Mann–Whitney U-test (for age, length of stay and number of complications) or the Fisher’s exact test (gender ratio, number of patient without any complications, re-surgery for complication, type of anaesthesia used and final outcome) and a p-value of less than 0.05 was considered to indicate statistical significance.

## Results

A total of 372 patients were enrolled in the study, out of which 200 patients were in control group and 172 patients were in the study group. In control group 101 were emergency surgeries and 99 were elective surgeries. In the study group, 80 and 92 were the emergency and the elective surgeries, respectively.

There were no significant differences in age, sex, type of anesthesia and mean length of hospital stay (Table 1). The total number of complications in control group was 403, out of which 239 complications occurred in emergency cases and 164 complications in elective cases. The total number of complications in the study group was 231, out of which 135 complications occurred in emergency cases and 96 occurred in elective cases (Table 2).

**Table 1:** Characteristics of patients.

Characteristics	Elective case		p-value	Emergency case		p-value
	Control group	Study group		Control group	Study group	
No. of patients	99	92		101	80	
Male sex (%)	62.62	51.08	0.11	83.16	80.0	0.69
Mean age (yr)	47.83	42.42	0.79	42.11	44.25	0.79
Mean length of hospital stay (days)	12.60	15.04	0.12	9.95	10.02	0.71
Anaesthesia GA (%)	78.80	84.80	0.76	51.50	53.8	0.47

**Table 2:** Complications in each group.

	Elective cases (n = 191)			Emergency cases (n = 181)		
	Control group (n = 99)	Study group (n = 92)	p-value	Control group (n = 101)	Study group (n = 80)	p-value
Total number of patients developed complications	66	47		78	54	
Number of complications	164	96	0.008	239	135	0.024
Total re-surgeries	05	Nil	0.06	06	06	1.00
Mortality	01	01	1.00	07	07	1.00

Nature of the complications and their total number in each group is given in Table 3. Surgical site infection and respiratory complications were more common among other complications (Table 3). Total re-surgeries because of complications were 11 (six in emergency, five in elective surgeries) and six (all in emergency) in control and study group, respectively. Total mortalities encountered were eight in each group.

**Table 3:** Nature and number of complications in both the groups. * SSI: Surgical site infection

	Elective cases			Emergency cases		
Complications	Control group N = 99	Study group N = 92	p-value (Binomial proportion double sided)	Control group N = 101	Study group N = 80	p-value (Binomial proportion double sided)
SSI *	38	27	0.047	54	30	0.0009
Respiratory system	30	24	0.32	41	24	0.02
Lower respiratory infection	15	9		30	13	
Upper respiratory infection	6	4		6	5	
Others	9	11		5	6	
Cardiac system	10	6	0.14	8	3	0.02
Atrial fibrillation	6	4		3	2	
Others	4	2		5	1	
Renal system	14	2	<0.0001	39	20	0.001
Acute kidney injury	5	1		28	18	
Others	9	1		11	2	
Surgical site collection	15	8	0.020	15	10	0.47
Hematoma	3	1		4	1	
Seroma	12	7		11	9	
Neurological	1	1	0.99	8	5	0.47
Delirium	-	1		7	5	
Other	1	-		1	-	
Sepsis	8	6	0.52	25	13	0.019
Gastrointestinal system	27	12	0.0002	33	21	0.13
Anastomotic leak	3	3		2	3	
Collection without leak	8	4		19	17	
Burst abdomen	4	1		5	6	
Ileus	12	4		7	5	
Stoma related	4	NIL		2	3	0.35
Refractory electrolyte Imbalance	5	2	0.04	7	3	0.08
Bleeding	4	1	0.002	2	1	0.47
Sub cutaneous emphysema	4	2	0.17			
Flap necrosis at surgical site	3	2	0.51	NIL	1	
Other	1	3		5	1	
	164	96	0.008	239	135	0.024

The percentage of patients developed complication in control and study group was 66.66% and 51.09% respectively for elective surgeries and 77.23% and 67.5% for emergency surgeries.

## Discussion

SURPASS checklist is the first valid checklist that follows the entire surgical pathway of the patient from admission to discharge. This multidisciplinary checklist was developed by de Vries et al. in 2008 and evaluated in high-performance hospitals of the Netherlands [2,5]. We have conducted a prospective clinical study in our hospital to see the effect of SURPASS checklist on patients’ outcomes in our hospital.

In our study, when the SURPASS checklist was implemented on both elective and emergency surgeries, a statistically significant reduction of postoperative complications was evident (p-value 0.008 in elective and 0.024 in emergency cases). But there was no significant difference in mortality rate after the implementation. The total numbers of complications in the control group were 404 among a total of 200 patients and 231 among a total of 172 patients in the study group which was statistically significant.

Our study showed that the implementation of SURPASS checklist was effective in preventing most of the postoperative complications. Most common postoperative complication seen in our study in both elective and emergency procedures was surgical site infection, which significantly came down after using this checklist which may be because of the proper preoperative administration of antibiotics. Other major postoperative complications that reduced significantly in elective cases were kidney-related complications, surgical site collection, refractory electrolyte imbalance, intra-abdominal complications (anastomotic leak, ileus, intra-abdominal collection) and bleeding. In emergency cases, other complications like respiratory, cardiac and kidney-related complications, and sepsis were also significantly reduced.

In elective cases, we found that 66.6% of patients developed complications in control group and it was reduced to 51.1% after implementation of the checklist in the study group that was statistically significant (p-value = 0.039). Whereas in emergency cases, 77.2% of patients developed complications before implementation of the checklist, and it was reduced to 67.5% in the study group, but the result was not statistically significant (p-value = 0.178).

This improvement in the rate of postoperative complications, after implementation of SURPASS checklist, can be explained by many factors. This checklist follows the full surgical pathway of the patient from admission to discharge and is responsible for providing additional care to the patient at all the level by implementing changes in the health care system, work pattern and safety behaviour of members of the surgical team. While using this checklist all the items of each part of checklist has to be verified by a responsible person and during checking off these items many critical things can be corrected prior to surgery, for example, timely cessation of anticoagulants, arrangement of postoperative ICU bed, recognition of medications patient may be allergic to, arrangement of all data related to patient’s previous problems, imaging data and laboratory results. In operation theatre, implementation of ‘time in’ component just before surgery is to verify the identity of the patient and surgical site, checking the availability of all required instruments and equipment, timely administration of premedication and most importantly timely administration of appropriate antibiotics. This formal pause just before the surgery is responsible for the significant reduction in the rate of the number of complications after surgery [6,7]. Timely arrangements of the instruments and equipment are associated with less technical problems and less delay in the time of surgery [8]. SURPASS checklist encourages not only correct antibiotic but the timely administration of antibiotics which is one of the important factors in the prevention of postoperative infections [9].

Due to the implementation of SURPASS checklist, the safety behaviour of the surgical team and their performance improvement occurs due to the fear of knowledge of being observed, which is known as the Hawthorne effect [10]. Many factors and items on the checklist are responsible for the improvement of safety behaviour of the surgical team towards patient care. Factors such as counseling the patients regarding the procedure and the likely complications during and in the postoperative period by the operating surgeon, counseling regarding anaesthesia and its complications by anesthesiologist, preparation of the patient before shifting to OT by ward nurse, marking the operative site, timely administration of appropriate antibiotics and ICU bed arrangement by ward doctor, all these make a definitive positive implication in proper patient management.

In such studies, confounding factors such as the age and sex of the patient, types of cases and surgery performed, type of anaesthesia used, could cause problems during statistical analysis. But in our study, there is no significant difference in these factors in both the groups. One more important confounding factor is the place or hospital where the effect of implementation of the checklist is being studied. In a study, the checklist that was compiled by World Health Organisation (WHO) was being implemented in different hospitals of different countries, many of them showed no significant effect of implementation of the checklist, but final overall results were statistically significant [11]. But in our study, we have included all patients of a single surgical unit from the Department of Surgery and also we have included both elective and emergency surgeries in equal numbers.

If we compare our study with a similar type of study done by de Vries et al. [5] in the Netherlands, effectiveness of comprehensive surgical safety system (SURPASS checklist) on patient outcomes showed significant reduction both in the rate of postoperative complications and mortality rate but in our study showed there is significant reduction only in the rate of postoperative complications, but not in mortality.

Failure in technical performance during surgery is a major cause of operative complications and adverse events. Unavailability of relevant medical data of the patient, lack of all investigation and important imaging data in OT are the major cause of poor technical performance. By using SURPASS checklist, these technical problems can be corrected which improves patients outcome [2,12]. Preoperative visit of the surgeon in the ward to see patient and instructions related to the surgery to the patient and ward doctor are also responsible for the improved patient outcome. Timely cessation of anticoagulants especially in vascular surgery and major procedures is responsible for the decrease in postoperative bleeding, which can be done by using this checklist [13]. Postoperative instructions by the surgeons and anesthesiologist regarding analgesia, fluid management, drains and oxygen and ventilator setting with proper follow-up of these instructions are responsible for the increase in improved patient outcomes, which can be carried out by using this checklist.

There are few limitations in our study. First, the small sample size—documentation of complications was limited to the duration of hospital stay of the patient and the duration of surgery was not documented which is an important risk factor for postoperative surgical site infection and other complications [14,15]. One more limitation of the study was the duration of symptom in emergency cases was not included, which some studies prove is an important risk factor for the postoperative outcome [15].

## Conclusions

Our clinical study showed that this multidisciplinary and comprehensive SURPASS checklist is effective in reducing the rate of postoperative complications in both elective and emergency surgeries, but there is no effect of implementation of the checklist on the rate of postoperative mortality.
